# Tanshinone II A enhances pyroptosis and represses cell proliferation of HeLa cells by regulating miR-145/GSDMD signaling pathway

**DOI:** 10.1042/BSR20200259

**Published:** 2020-04-15

**Authors:** Wenjuan Tong, Jianghong Guo, Chunfen Yang

**Affiliations:** Department of Obstetrics and Gynecology, The First Affiliated Hospital, University of South China, Hengyang, Hunan, China

**Keywords:** cell proliferation, cervical cancer, GSDMD, miR-145, pyroptosis, tanshinone II A

## Abstract

Cervical cancer is the fourth most common cancer in women globally. Lack of effective pharmacotherapies for cervical cancer mainly attributed to an elusive understanding of the mechanism underlying its pathogenesis. Pyroptosis plays a key role in inflammation and cancer. Our study identified microRNA (miR) 145 (miR-145)/gasdermin D (GSDMD) signaling pathway as critical mediators in the effect of tanshinone II A on HeLa cells. In the present study, we found that treatment of tanshinone II A led to an obvious repression of cell proliferation and an increase in apoptosis on HeLa cells, especially in high concentration. Compared with the controlled group, tanshinone II A enhanced the activity of caspase3 and caspase9. Notably, the results demonstrated that tanshinone II A regulated cell proliferation of HeLa cells by regulating miR-145/GSDMD signaling pathway. Treatment of tanshinone II A significantly up-regulated the expression of GSDMD and miR-145. After transfection of si-miR-145 plasmids, the effects of tanshinone II A on HeLa cells were converted, including cell proliferation, apoptosis and pyroptosis. In addition, the results showed that tanshinone II A treatment altered the expression level of PI3K, p-Akt, NF-kB p65 and Lc3-I. Collectively, our findings demonstrate that tanshinone II A exerts anticancer activity on HeLa cells by regulating miR-145/GSDMD signaling. The present study is the first time to identify miR-145 as a candidate target in cervical cancer and show an association between miR-145 and pyroptosis, which provides a novel therapy for the treatment of cervical cancer.

## Introduction

Cervical cancer is ranked as the fourth most common cancer among women universally [1], yet it remains the second most common cause for cancer incidence in developing countries [2]. According to a research, there was an estimated 570000 new cases and 311000 deaths worldwide caused by cervical cancer in 2018, especially in low- and middle-income countries [3]. Unfortunately, extensive access to vaccination and screening have not achieved in the developing world, where 87% of all cervical cancer-related deaths occur [1,4]. Currently, cisplatin, with concurrent radiotherapy, is the primary cytotoxic agent used to treat patients with advanced cervical cancer, which is regarded as the standard treatment of cervical cancer [5–8]. However, cervical cancer showed chemotherapy-resistant effect in several researches. The responses with single-agent cisplatin are often not durable, and the objective response rate (ORR) only ranges from 13 to 23% [9,10]. In light of these findings, additional studies to investigate more treatment strategies of cervical cancer are imperative.

As a lipophilic pharmacologically active compound extracted from *Salvia miltiorrhiza Bunge* (Danshen), tanshinone II A shows a variety of biological activities, including anti-inflammatory [11–13], antibacterial [14], antitumor [15], antioxidative [16], antimutagenic [17] and antiplatelet aggregation activities [18]. Several researches revealed that tanshinone II A inhibited lipopolysaccharide (LPS)-induced acute lung injury in mice by regulating PI3K/AKT and MAPK signaling, and ameliorated the cardiovascular dysfunction by regulating NADPH oxidase 2-related signaling [19]. Moreover, it is widely accepted that tanshinone II A is able to prohibit malignant proliferation and to reinforce malignant cell death, and significantly eliminates malignant cells. Tanshinone II A treatment can repress HPV E6 and E7 oncogenes, and reactivates p53-dependent tumor suppressor, which leads to a growth inhibition of cervical cancer cells [20]. However, the underlying molecular mechanisms by which Tanshinone II A inhibited cervical cancer cell proliferation are still elusive.

It is well known that pyroptosis is an inflammatory form of programmed cell death [21], and typically characterized by cellular swelling, pore formation in the membrane, cell lysis and release of pro-inflammatory molecules [22]. Recent studies have demonstrated that inducement of pyroptosis is accompanied by NF-kB inhibition in the inflammation [23,24]. Although tanshinone II A can attenuate invasion and metastasis of cancer cells by blocking NF-kB activation, little is known about the role of pyroptosis inhibited by tanshinone II A in cervical cancer.

MicroRNAs (miRs) are known as a family of non-coding RNAs with a length of 18–24 nt, which participates in almost all the developmental and progression processes [25]. In tumors, miRs can either function as tumor suppressor gene or oncogene by regulating downstream targets. It is reported that miR-145 was down-regulated in nasopharyngeal carcinoma [26], papillary thyroid carcinoma [27] and head and neck cancers [28]. For example, Liu et al. [26] reported that miR-145 directed with CASC9, and the inhibition of miR-145 promoted cell migration and invasion but inhibited cell apoptosis in nasopharyngeal carcinoma cells, which provided a novel therapy for the treatment of nasopharyngeal carcinoma. Furthermore, there are various researches showing a robust association between miR-145 and inflammation [29–31]. However, the role of miR-145 in the treatment of tanshinone II A to cervical cancer have not been fully elucidated.

In the present study, we found that tanshinone II A repressed the proliferation and inflammation on HeLa cells, which was associated with increased level of pyroptosis and apoptosis. It is worth noting that down-regulation of PI3K/AKT signaling was also identified in the study. Additionally, we studied the potential targets of miR-145 and the effect of miR-145 on tanshinone II A-treatment to protect against cervical cancer. Our study may provide new insights for the therapy of cervical cancer.

## Materials and methods

### Cell culture and chemicals

**Figure 1:**
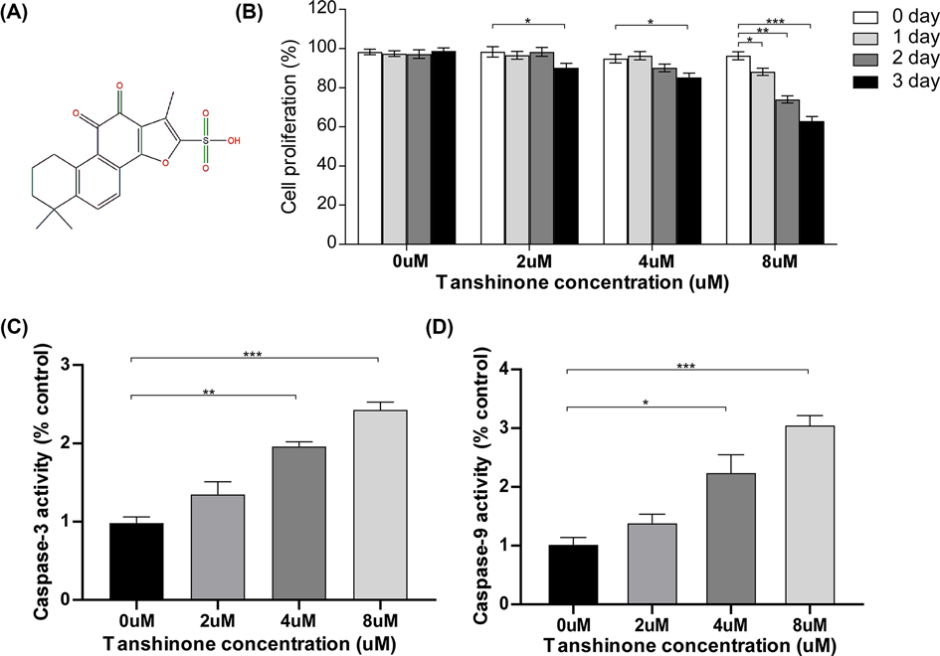
Effect of tanshinone II A on the apoptosis on HeLa cells (**A**) Chemical structure of Tanshinone II A. (**B**) Cell proliferation rate at different concentrations (0, 2, 4, 8 μm) and different time (24, 48, 72 h) by using MTT assay after administration of Tanshinone II A. *P<0.05, **P<0.01, ***P<0.005. (**C,D**) ELISA analysis of the caspase9 and caspase3 activity in HeLa cells. * *P*<0.05, **P<0.01, ***P<0.005.

Human cervical cancer cell line HeLa was kindly gifted by Dr. Nie (China-Japan Friendship Hospital, China). HeLa cells were cultured in standard medium which includes DMEM (C11995500BT; Gibco), 10% FBS (P30-3301; Pan) and 1% penicillin–streptomycin (15140-122; Gibco). Cells were cultured in 5% CO_2_ under a water-saturated atmosphere in a cell incubator at 37°C. Tanshinone II A (Figure 1A) was procured from Sigma–Aldrich, St. Louis, MO, U.S.A. (51704-10MG; Sigma). DMSO (276855, Sigma) was used as negative control. MTT kit was purchased from Solarbio (M1020).

### Cell transfection

The miR-145 mimic and its negative control were purchased from RiboBio (Guangzhou, China). The HeLa cells were cultured in six-well plates at a density of 1 × 10^7^ cells/ml for 24 h. According to the manufacturer’s instructions, Lipofectamine 2000 reagent (11668-027, Invitrogen) was used for transfection. Cells were harvested for further administration after transfection for 48 h.

### MTT assay

HeLa cells (1 × 10^6^ cells/well) were seeded and cultured in six-well plates for 12 h. Subsequently, HeLa cells were exposed to different concentrations of Tanshinone II A (0, 2, 4 and 8 µM) for 0, 24, 48 and 72 h. A total of 20 µl of MTT solution was added to each well and incubated 5% CO_2_ under a water-saturated atmosphere in a cell incubator at 37°C. Furthermore, the medium was entirely removed, following 500 μl of DMSO administration. In the end, ELISA plate reader was used to determinate the optical density (OD).

### Caspase3 and caspase9 activity assay

HeLa cells (1 × 10^6^ cells/well) were cultured in six-well microplates, and cells were seeded overnight at 37°C in a 5% CO_2_ incubator. Then, the HeLa cells were administered with different concentrations of Tanshinone II A (0, 2, 4 and 8 µM) for 48 h. Cell lysis buffer (R0020, Solarbio) was used to extract all protein from the HeLa cells. After that, lysates were cleared by centrifugation, following with a measurement of protein concentration by BCA protein assay kit (P1511-1, Applygen). Caspase3 ELISA Kit (BC3830, Solarbio) and caspase9 (BC3890, Solarbio) ELISA Kit were used according to the manufacturer’s instructions. The absorbance values were determined at 405 nm using the ELISA plate reader. The activity levels were expressed compared with the control group.

### Western blot analysis

First, HeLa cells were lysed in RIPA lysis buffer (R0020, Solarbio), which contains 1% protease inhibitor cocktail (B14001; Bimake) and 1% phosphatase inhibitor (B15001; Bimake). The BCA Assay Kit (P1511-1, Applygen) was used to determined the protein concentration of lysate. Proteins were separated on 10% SDS/PAGE gels and then transferred to PVDF membranes (IPVH00010; Millipore). The membranes were blocked for 2 h at room temperature in Tris-buffered saline and 0.1% Tween-20 (TBST) containing 5% skim milk and then were incubated with primary antibodies in the same buffer at 4°C overnight. The protein bands were detected with HRP–conjugated secondary antibodies and Clarity Western ECL Substrate (170-5060; Bio-Rad). The protein bands were visualized with a Bio-Rad System (Bio-Rad, Hercules, CA, U.S.A.). Actin served as a loading control.

### Antibodies

Antibodies against p-Akt (sc-7985-R), Akt (sc-1618), PI3K (sc-67306), NF-kB (sc-514451) were obtained from Santa Cruz Biotechnology (Santa Cruz, CA, U.S.A.). Antibodies specific for LC3 (ab51520), gasdermin D (GSDMD; ab210070), IL18 (ab191152), IL1b (ab9722) were purchased from Abcam (Cambridge, MA, U.S.A.). The actin antibody (ab-2768234) was produced by ABclonal (Wuhan, China). The dilution ratio for all primary antibodies was 1:2000. The secondary antibodies used in the present study were peroxidase AffiniPure goat anti-rabbit-IgG (H+L) (BF03008X) and goat anti-mouse-IgG (H+L) (BF03001X), which were obtained from Bio Dragon (Beijing, China). Secondary antibodies were used at 1:5000 dilution.

### Quantitative reverse-transcription PCR

Real-time PCR (qPCR) was performed as standard protocol. Briefly, total RNA was isolated from HeLa cells using TRIzol reagent (15596-026; Invitrogen). Subsequently, the total RNA was reverse-transcribed into cDNA using a Transcriptor First-Strand cDNA Synthesis Kit (04896866001; Roche) according to the manufacturer’s instructions. The ABI-Quant studio 5 system and the SYBR Green (TransGen, Beijing, China) detection format were used to quantify the PCR amplification products. The mRNA expression levels of the target genes were normalized to *β-actin* expression. The primer pairs used in the present study are listed in Supplementary Table S1.

### Hoechst/PI staining

HeLa cells in six-well plates were washed twice with cold PBS and then incubated in 1 ml of binding buffer consisting of 0.5 ml of Hoechst solution and 0.5 ml of PI solution. After incubating at 4°C for 30 min, the cells were washed twice with PBS and analyzed with an inverted fluorescence microscope. Viable cells were both Hoechst- and PI-negative and apoptotic cells were Hoechst-positive PI-negative, while necrotic cells were both Hoechst- and PI-positive.

### Statistical analysis

All results are presented as means ± SD, further analyzed by using the appropriate statistical analysis methods, as specified in the figure legends, with SPSS software (version 24.0). For data that showed a normal distribution and homogeneity of variance, a two-tailed Student’s *t* test was used to compare differences between two groups. One-way ANOVA was applied for multiple comparisons, followed by Tukey’s post hoc analysis (for data showing homogeneity of variance). Data from all studies were collected in a blinded fashion. No data were excluded when performing the final statistical analysis. A randomization process was performed in grouping mice with the same phenotypes. *P*<0.05 was considered statistically significant.

## Results

### Effects of tanshinone II A on the cellular proliferation and apoptosis of HeLa cells

First, we investigated the effects of tanshinone II A on the cellular proliferation of HeLa cells by using an MTT assay. HeLa cells were treated with a range of tanshinone II A concentrations (0, 2, 4 and 8 µM) for 0, 24, 48 and 72 h. The results showed that after administration of tanshinone II A, the proliferation of HeLa cells was inhibited in a dose- and time-dependent manner. Treatment with tanshinone II A (2, 4 and 8 µM) for 72 h or tanshinone II A (8 µM) for 24 and 48 h led to significant reductions in cellular proliferation (Figure 1B). Furthermore, we investigated the effects of tanshinone II A treatment on apoptosis in HeLa cells by detecting the activity levels of caspase9 and caspase3. As shown in Figure 1C,D, tanshinone II A enhanced the activity levels of caspase3 and caspase9 in HeLa cells in a dose-dependent manner. Administration with tanshinone II A (4 and 8 µM) resulted in a significant increase in the activity levels of caspase3 (Figure 1C) and caspase9 (Figure 1D). These results suggested that tanshinone II A exhibited obvious anticancer effects on human cervical cancer cells.

### Effects of tanshinone II A on the PI3K/Akt and NF-kB/LC3 expressions of HeLa cells

**Figure 2:**
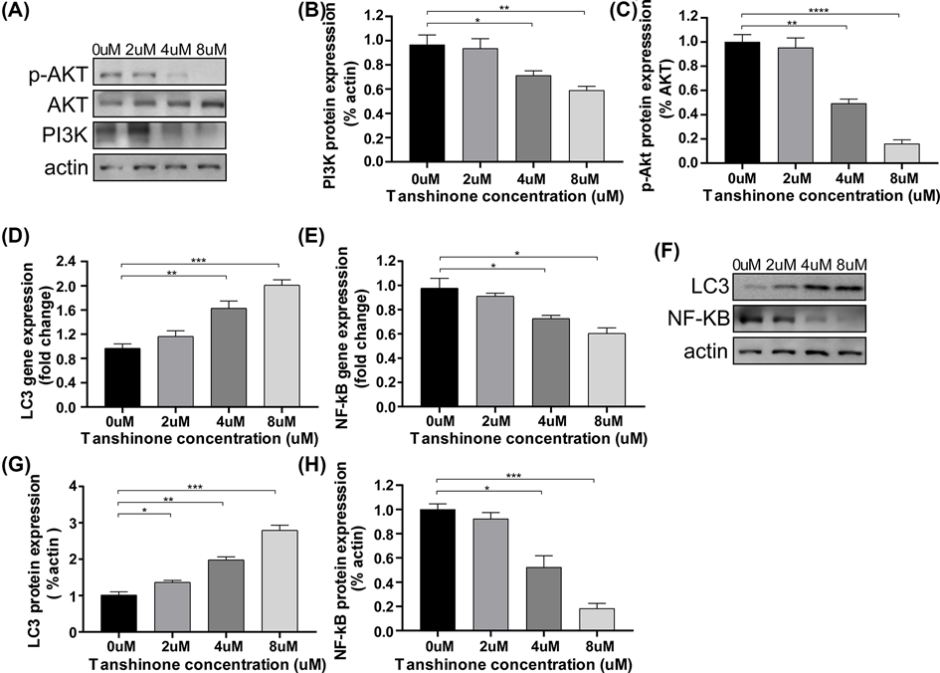
Effect of tanshinone II A on the regulation of Akt/PI3K and NF-kB/LC3 signaling on HeLa cells (**A**–**C**) Representative Western blot analysis (A) and quantification (B,C) of Akt, p-Akt and PI3K protein on HeLa cells at different concentrations (0, 2, 4, 8 μm) after treatment for 72 h. Protein expression was normalized to β-actin levels. *P<0.05, **P<0.01, ***P<0.005, ****P<0.001. (**D,E**) qPCR analyses of the relative mRNA levels of LC3 and NF-kB on HeLa cells after treatment for 72 h. Gene expression was normalized to β-actin mRNA level. *P<0.05, **P<0.01, ***P<0.005. (**F**–**H**) Representative Western blot analysis (F) and quantification (G,H) of LC3-I and NF-kB p65 protein on HeLa cells after treatment for 72 h. *P<0.05, **P<0.01, ***P<0.005, ****P<0.001.

The PI3K/Akt and the NF-kB/LC3 signaling pathway are both involved in the critical physiological process in cancer cells. To confirm whether tanshinone II A could regulate the protein expressions of PI3K/Akt and NF-kB/LC3 on HeLa cells, qPCR and Western blotting assay was carried out. Notably, compared with the untreated cells, tanshinone II A administered cells showed a down-regulation of PI3K/Akt expressions (Figure 2A–C). In addition, the results also showed that treatment of tanshinone II A decreased the NF-kB p65 expression, which was accompanied with an up-regulation of LC3-I (Figure 2D–H), especially in the concentrations of 4 and 8 µM. Henceforth, the results indicated that tanshinone II A promoted autophagy on HeLa cells by regulating NF-kB/LC3 signaling, and the anticancer effects of tanshinone II A were also associated with modulation of PI3K/Akt signaling pathway.

### Effects of tanshinone II A on the pyroptosis of HeLa cells

**Figure 3:**
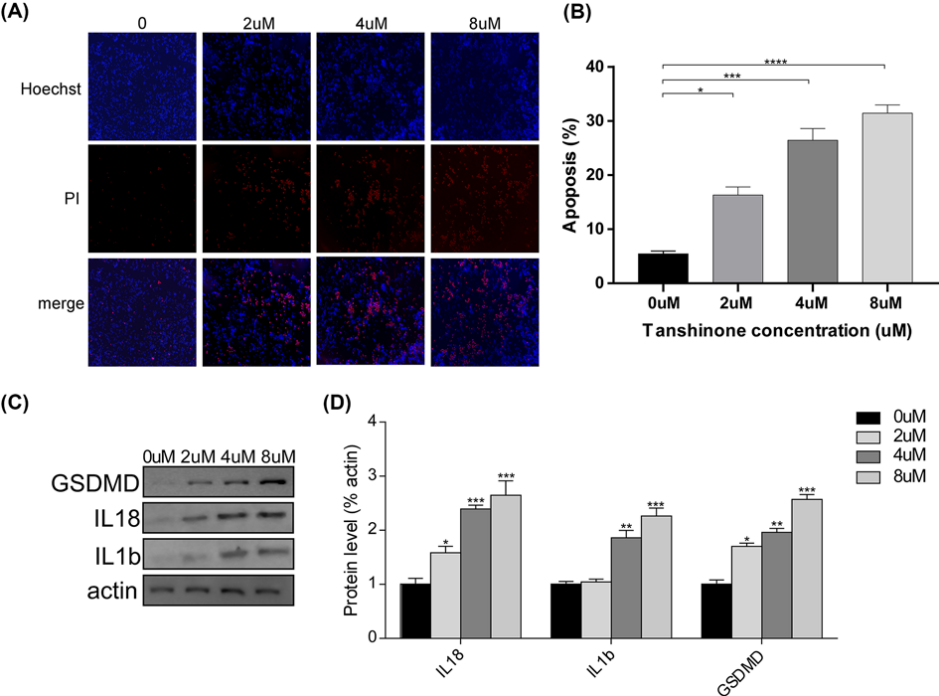
Effect of tanshinone II A on the pyroptosis on HeLa cells (**A,B**) Hoechst-PI staining (A) and quantification (B) of apoptosis on HeLa cells at different concentrations (0, 2, 4, 8 μm) after treatment for 72 h. *P<0.05, **P<0.01, ****P<0.001. (**C,D**) Representative Western blot analysis (C) and quantification (D) of GSDMD, IL18 and IL1b protein on HeLa cells after treatment for 72 h. *P<0.05, **P<0.01, ***P<0.005.

From apoptosis-inducing properties of tanshinone II A, we speculated that it might lead to high level of pyroptosis. Hence, Hoechst-PI staining was carried out to detect the level of pyroptosis. As expected, the results revealed that tanshinone II A enhanced pyroptosis of HeLa cells at the concentrations of 2, 4 and 8 µM (Figure 3A,B). Moreover, GSDMD expression was also up-regulated after treatment of tanshinone II A as compared with untreated cells (Figure 3C,D). We also detected the expression level of IL-18 and IL-1b, which is positively associated with GSDMD [32]. We observed that the expression of IL-18 and IL-1b were both increased after administration of the tanshinone II A (Figure 3C,D). The results suggested that tanshinone II A is an effective molecule for activating pyroptosis in HeLa cells.

### Role of miR-145 on the effects of tanshinone II A on HeLa cells

**Figure 4:**
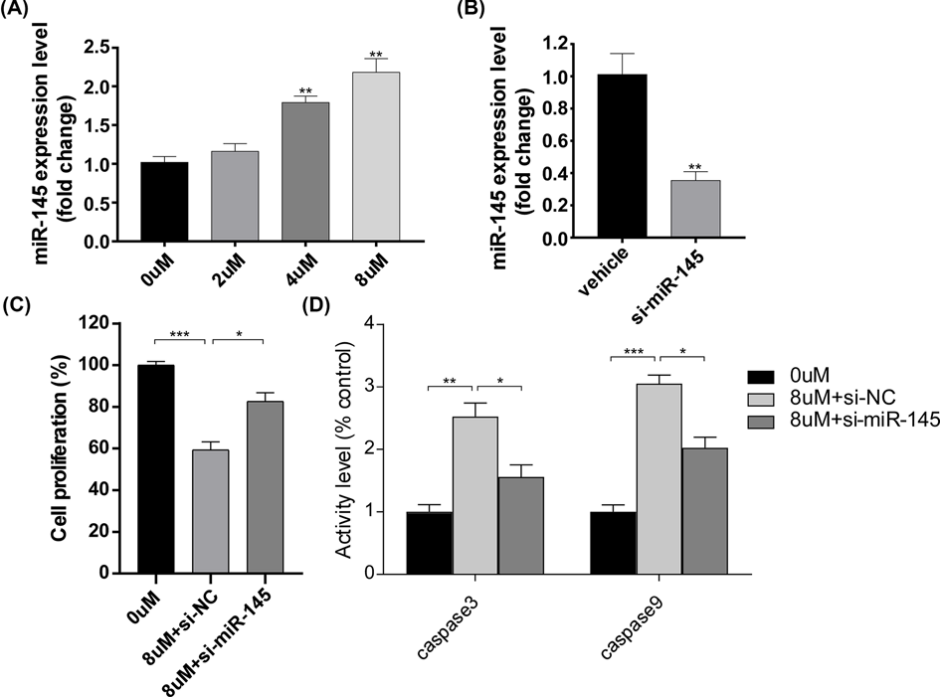
Role of miR-145 in the effect of Tanshinone II A on HeLa cells (**A**) qPCR analyses of the relative mRNA levels of miR-145 on HeLa cells at different concentrations (0, 2, 4, 8 μm). Gene expression was normalized to U6 mRNA level. *P<0.05, **P<0.01. (**B**) The mRNA levels of miR-145 after transfection of si-miR-145 plasmids. *P<0.05, **P<0.01. (**C**) Cell proliferation rate after plasmids transfection and Tanshinone II A treatment for 72 h. *P<0.05, **P<0.01, ***P<0.005. (**D**) ELISA of caspase3 and caspase9 activity after plasmids transfection. *P<0.05, **P<0.01, ***P<0.005.

**Figure 5:**
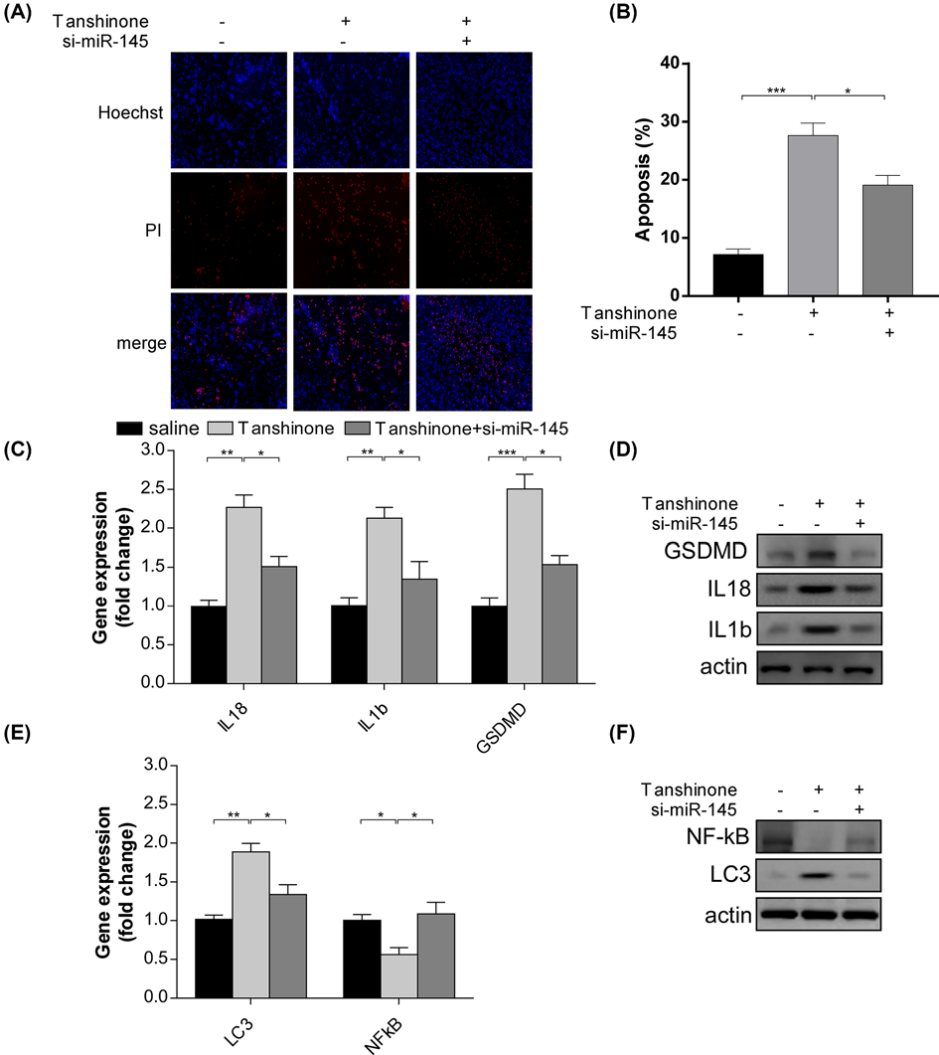
Tanshinone II A enhanced apoptosis and pyroptosis by regulating miR-145 on HeLa cells (**A,B**) Representative images (A) and quantification (B) of Hoechst-PI staining after transfection of si-miR-145 plasmids. *P<0.05, ***P<0.005. (**C,D**) qPCR analysis (C) and immunoblotting assay of GSDMD, IL18 and IL1b. *P<0.05, **P<0.01, ***P<0.005. (**E,F**) qPCR analysis (C) and immunoblotting assay of LC3-I and NF-kB p65 after transfection of si-miR145 plasmids. *P<0.05, **P<0.01.

As previous researches have shown, miR-145 was down-regulated in various types of cancer. Therefore, we investigated whether miR-145 was involved in the regulatory process induced by tanshinone II A. Notably, we found that the expression of miR-145 was increased after the treatment of tanshinone II A for 72 h (Figure 4A). Furthermore, anti-miR-145 plasmids were transfected into HeLa cells. Compared with vehicle group, the anti-miR-145 plasmids significantly reduced the expression levels of miR455-3p in HeLa cells (Figure 4B). In addition, the anti-miR-145 plasmids were able to convert the effect of tanshinone II A on HeLa cells, such as cell proliferation (Figure 4C) and the activity level of caspase3 and caspase9 (Figure 4D). To further analyze the role in the effect of tanshinone II A on HeLa cells, we detected the level of pyroptosis after the treatment of tanshinone II A and the transfection of anti-miR-145 plasmids. It is worth noting that compared with vehicle group, anti-miR-145 plasmids significantly reversed the level of pyroptosis induced by tanshinone II A (Figure 5A,B), which was accompanied with a down-regulation of the protein level of GSDMD, IL-18 and IL-1b (Figure 5C,D). Moreover, we also investigated the expression of LC3-I and NF-kB p65 after the administration of anti-miR-145 plasmids. By using qPCR and immunoblotting analysis, we found that transfection of anti-miR-145 plasmids led to a decrease in LC3-I expression and an up-regulation of NF-kB p65 protein (Figure 5E,F), suggesting that miR-145 was involved in the autophagy progress regulated by tanshinone II A. The results demonstrated that miR-145 plays an important role in the effect of tanshinone II A on HeLa cells.

## Discussion

Cervical cancer is one of the widespread leading causes among women around the world. At present, almost all targeted therapies are hard to improve the outcomes of cervical cancer, which has approximately 60% mortality in 5 years [33]. Therefore, it is necessary to investigate the potential drug for developing effective therapy and explore the underlying molecular mechanisms of cervical cancer. As a lipophilic natural compound extracted from Danshen, tanshinone II A has been reported to exert anticancer activity on cervical cancer [34]. However, the specific molecular mechanism has not been evaluated yet. In the present study, we investigated the mechanism underlying the anticancer effects of tanshinone II A on human cervical cancer cells. The results presented here provide evidence that modulation of miR-145/GSDMD is closely involved in the anticancer activity of tanshinone II A on HeLa cells, which provides a strong evidence to support that tanshinone II A can act as a potential anticancer drug against cervical cancer.

As a critical form of cell death, pyroptosis has been reported to be involved in many physiological processes, such as regulation of tumor immune microenvironment [35], treatment of acute myeloid leukemia [36] and act as an innate immune effector against intracellular bacteria [37]. There are various researches showing an association between pyroptosis and tumor proliferation [38–40]. In this study, we found that tanshinone II A enhanced cell proliferation and inhibited apoptosis by up-regulating GSDMD expression on HeLa cells, especially at high concentrations. These results were similar to So et al.’s [41] findings, which showed representative features of pyroptosis after sirt1 knockdown in cervical cancer cells. Furthermore, the results also showed that tanshinone II A was able to decrease the expression of NF-kB p65. This finding was consistent with the previous study showing that tanshinone IIA inhibited LPS-induced IkBa degradation and NF-kB activation in RAW 264.7 cells, which provided a potent evidence of the anti-inflammatory activity of tanshinone IIA [42]. It has been shown that inducible activation of NF-kB with LPS promoted IL1b expression. However, in the present study, we found that compared with control group, the administration of tanshinone II A up-regulated the expression of IL18 and IL1b, which might attribute to the increased activity of caspase1. Pyroptosis is regulated via a caspase1-dependent mechanism [32]. Caspase1-dependent pyroptosis requires activation of the canonical inflammasomes. In this pathway, caspase1 directly cleaves GSDMD and the precursor cytokines pro-IL1β and pro-IL18, initiating pyroptosis and maturation of IL1β and IL18, respectively. The cleaved N-terminal portion of GSDMD forms pores on the host cell membrane to mediate the release of cytoplasmic contents. In the present study, we found that the caspase1 expression was significantly up-regulated after tanshinone II A administration (data not shown), which contributed to high levels of IL1b and IL18. These results provided a strong evidence to support that tanshinone II A might exert anticancer activity by regulating pyroptosis on HeLa cells.

miRNA is a type of non-coding small RNA molecule, which has emerged as a critical molecular marker in development and progression of many cancers [43,44]. For instance, silencing of miR-484 could aggravate the malignancy of cervical cancer cells by inhibiting MMP14 and HNF1A [43]. In addition, it was observed that exosomal miR-106b increased the MMP-2 and MMP-9 expression, leading to an enhancement of the migration and invasive ability of BEAS-2B cells [44]. miR-145 has been reported in various researches. According to a recent study, miR-145-1 expression was significantly decreased in cervical cancer cell tissues and cell lines. The overexpression of miR-145 led to an inhibition of cell proliferation, migration and invasion in cervical cells. Additionally, the miR-145 agomir was able to repress WNT2B expression, and further led to an inhibition of the Wnt/β-catenin signaling in cervical cancer cells [45]. Another research also demonstrated that miR-145 could target c-myc which interacted physically with DNMT3A in ovarian cancer cells, and inhibit the Warburg effect through miR-133b/PKM2 pathways [46]. Similar to previous studies, our results revealed that tanshinone II A increased the expression of miR-145 at mRNA level on HeLa cells. The level of cell proliferation and apoptosis was converted after transfection of si-miR-145 plasmids. Moreover, si-miR-145 plasmids also inhibited the GSDMD expression at protein and mRNA levels. In addition, we found that compared with the group with single treatment of tanshinone II A, transfection of si-miR-145 plasmids led to a decreased expression of IL18, IL1b and caspase1 as well as an up-regulation of NF-kB, which suggested that si-miR-145 plasmids also influenced the autophagy activity on HeLa cells.

In conclusion, our study demonstrated that tanshinone II A may be regarded as a potential candidate for the treatment of cervical cancer by regulating miR-145/GSDMD signaling pathway. The administration of tanshinone II A inhibited cell proliferation of HeLa cells by up-regulating miR-145 and GSDMD. Transfection of si-miR-145 plasmids reversed the effect induced by tanshinone II A. It is the first time to regard miR-145 as a candidate target in cervical cancer and demonstrated an association between miR-145 and pyroptosis. With limited drug options available for ovarian cervical cancer and limited toxicity, tanshinone II A represents a strong option for therapy of cervical cancer.

## Supplementary Material

Supplementary Table S1

## Abbreviations

CASC9: cancer susceptibility candidate 9
DNMT3A: DNA methyltransferase 3Α
GSDMD: gasdermin D
HNF1A: hepatocyte nuclear factor 1 A
HPV: Human papillomavirus
HRP: Horseradish Peroxidase
IkBa: I-kappa-B-alpha
IL: interleukin
LPS: lipopolysaccharide
miR: microRNA
MMP: matrix metalloproteinase
PI: propidium iodide
PKM2: pyruvate kinase M2
qPCR: real-time PCR
RIPA: Radio Immunoprecipitation Assay
sirt1: Silencing regulatory protein 1
WNT2B: wingless-type MMTV integration site family, member 2B
