# Supplementary material for: Tanshinone II A enhances pyroptosis and represses cell proliferation of HeLa cells by regulating miR-145/GSDMD signaling pathway
Source: Biosci Rep. 2020 Apr 15;40(4):BSR20200259. doi: 10.1042/BSR20200259 (PMC7160242; doi:10.1042/BSR20200259)
Supplement: Supplementary Table S1 [file BSR-2020-0259_supp.pdf]

Supplementary Table 1. Primers for quantitative real-time PCR detection.

Gene	Sequence
Lc3-FWD	5’-CGGCTTCCTGTACATGGTTT-3’
Lc3-REV	5’-AACCAT TGGCTTTGTTGGAG-3’
NF-kB-FWD	5’-GTGCTCGGTGGGAGTAAGAG-3’
NF-kB-REV	5’-CTCCCGTCACTGCATAGTCA-3’
miR-145-FWD	5’-CAGTGCGTGTCGTGGAGT-3’
miR-145-REV	5’-AGGTCCAGTTTTCCCAGG-3’
IL18-FWD	5’-CAGGCCTGACATCTTCTGCAA-3’
IL18-REV	5’-CTGACATGGCAGCCATTGT-3’
IL-1b-FWD	5’-AAGCCTCTCCACCTCCTCTC-3’
IL-1b-REV	5’-TTGTCCCTGATACCCAAGG-3’
GSDMD-FWD	5’-ATATCTGCCAGAGATTGATA-3’
GSDMD-REV	5’-TGGAAGTATCTTTGCCGGT-3’
Actin-FWD	5’-TGGACTTCGAGCAGGAAATGG-3’
Actin-REV	5’-ACGTCGCAC TTCATGATCGAG-3’
U6-FWD	5’-ATTGGAACGATACAGAGAAGATT-3’
U6-REV	5’-GGAACGCTTCACGAATTTG-3’
